# Association between the prevalence rates of circadian syndrome and testosterone deficiency in US males: data from NHANES (2011–2016)

**DOI:** 10.3389/fnut.2023.1137668

**Published:** 2023-05-09

**Authors:** Yunfei Xiao, Shan Yin, Jianwei Cui, Yunjin Bai, Zhenzhen Yang, Jiahao Wang, Jia Wang

**Affiliations:** ^1^Department of Urology, Institute of Urology, West China Hospital, Sichuan University, Chengdu, China; ^2^Department of Urology, Affiliated Hospital of North Sichuan Medical College, Nanchong, China; ^3^Department of Clinical Laboratory, Nanchong Central Hospital, Nanchong, China

**Keywords:** circadian syndrome, testosterone deficiency, NHANES, cross-section, association

## Abstract

**Objective:**

The objective of this study is to explore the association between the prevalence rates of circadian syndrome (CircS) and testosterone deficiency (TD).

**Materials and methods:**

Cross-sectional analysis was conducted on the basis of the National Health and Nutrition Examination Survey 2011–2016. The target population was males aged ≥20 years old. A total of three multivariable logistic regression models were built to elucidate the association between the prevalence rates of CircS and TD. Interactive and stratified analyses were employed to explore whether some variables can modify the above association. Sensitivity analyses were also conducted to verify the stability of the results with extreme values removed.

**Results:**

A total of 3,436 eligible participants were involved. Multivariable logistic regression in the fully adjusted model suggested the CircS group had a significantly higher prevalence rate of TD compared with the non-CircS group (OR = 2.284, 95%CI 1.569 to 3.323). No significant correlation between the number of CircS components and TD was observed in any of the three models. The interactive and stratified analyses showed the association was more obvious in the population with moderate or vigorous activities. The results were robust after extreme data were excluded.

**Conclusion:**

There is a positive association between the prevalence rates of CircS and TD in US men. The association becomes more obvious owing to moderate or vigorous activities.

## 1. Introduction

Testosterone, a sex hormone, is primarily secreted by testicular interstitial cells and slightly by adrenal glands and ovaries. Research certificates that testosterone concentrations are largely higher in men than in women (1). Testosterone not only plays a critical role in sex differentiation, growth, and development but also has multiple physiological functions. A substantial proportion of men in the United States (20%−50%) are affected by testosterone deficiency (TD) (2). TD has some negative impacts, such as psychophysiological diseases, sexual dysfunction, muscle atrophy, and cognitive disorders (3–6). Apart from being regulated by age and lifestyle habits, testosterone levels are modulated by circadian rhythms in synthesis and secretion. Clinical research shows that serum testosterone concentration is always accompanied by regular fluctuations in a 24-h cycle. Furthermore, the highest and lowest testosterone concentrations appear around fixed time points, indicating a circadian rhythm in testosterone secretion (7).

Previous studies have shown that the prevalence rates of metabolic syndrome (MetS) were positively associated with TD (8). In addition, MetS is often accompanied by sleep deprivation and depression, and the prevalence rates of TD increase with the onset of the above disorders. Recent studies report that circadian rhythm disturbances may be an essential etiology of MetS and its comorbidities (9). Thus, a novel concept called circadian syndrome (CircS) closely related to circadian rhythms emerged. CircS is primarily diagnosed based on hypertension, dyslipidemia, central obesity, diabetes, short sleep duration, and depression (10). Each of those symptoms is mainly governed by circadian rhythms, which are major regulators in almost every aspect of human health and metabolism. In addition to the associations with insulin resistance and cardiovascular diseases, CircS is often in conjunction with steatohepatitis, cognitive dysfunction, and lower urinary tract symptoms (10–12). Studies prove that modern individuals are more vulnerable to circadian disruption due to shift work, lack of exercise, excessive food intake, or exposure to light and noise (13–15). Therefore, we hypothesize that CircS may be an important potential cause of TD.

To the best of our knowledge, no research has been conducted focusing on the association between the prevalence rates of CircS and TD. Thus, this cross-sectional study has been conducted to explore this association by analyzing large population data from the National Health and Nutrition Examination Survey (NHANES).

## 2. Methods

### 2.1. Study design and population

**Figure 1:**
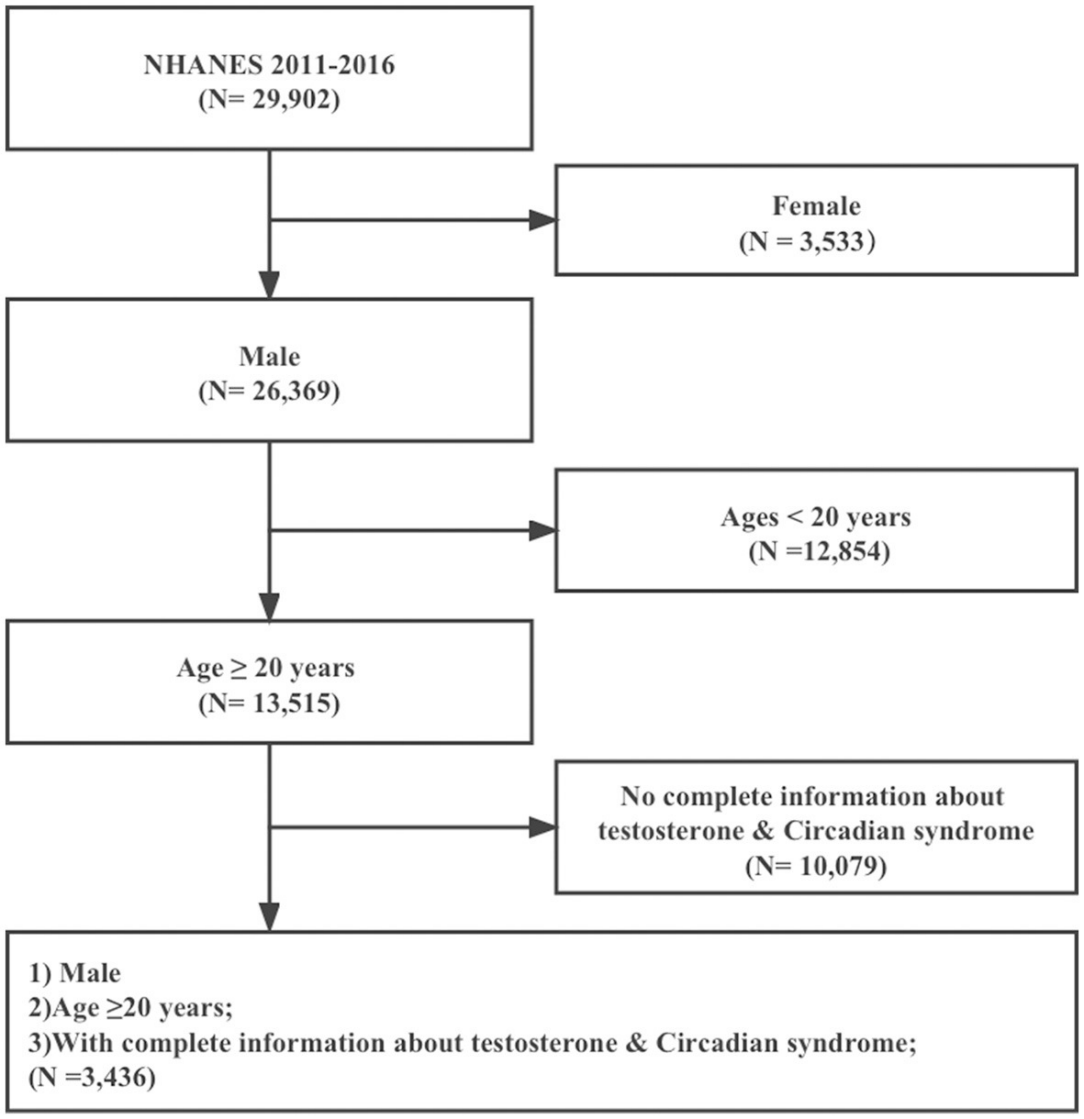
Flow diagram of obtaining the final inclusion in the population.

All of the data were collected from NHANES 2011–2016 (https://www.cdc.gov/nchs/nhanes/index.htm), which was designed by the National Center for Health Statistics (NCHS). NHANES is a nationally representative cross-sectional survey to measure the health and nutritional statuses of Americans and employs probability sampling at several stages to gather information from various sources, including interviews, physical exams, and laboratory tests. NHANES is accessible to researchers for free and has been updated every 2 years since 1999. This study initially involved 29,902 people. First, women were excluded (*n* = 3,533). Then, participants younger than 20 years old (*n* = 12,854) were removed. People with missing data for CircS diagnosis or testosterone (*n* = 10,079) were also excluded. Finally, a total of 3,436 participants were admitted (Figure 1).

All NHANES study protocols were approved by the Ethics Review Committee of the NCHS, and consent was obtained from all participants.

### 2.2. Diagnosis of the circadian syndrome

As the main exposure variable, CircS was diagnosed when a person had ≥4 of these components (16) as follows: (1) central obesity defined as men with a waist circumference of ≥102 cm, (2) elevated triglycerides (≥150 mg/dL) or use of lipid-lowering medication, (3) decreased high-density lipoprotein-cholesterol (< 40 mg/dL in men) or use of lipid-lowering medication, (4) elevated blood pressure (systolic ≥130 or diastolic ≥85 mmHg) or use of an antihypertensive drug, (5) elevated fasting glucose (≥100 mg/dL) or use of anti-diabetic medication, (6) reduced sleep duration (≤ 6 h/day), and (7) depression symptoms defined by patient health status questionnaire-9.

### 2.3. Testosterone deficiency and assessment of serum testosterone

Based on the guidelines of the American Urological Association (AUA), a condition characterized by TD was defined as a serum testosterone level below 300 ng/dL (17, 18). To minimize biological variability, serum specimens were collected in the morning after overnight fasting. After that, the specimens were shipped frozen on dry ice for immediate use or stored at −70°C in the long term. To measure total serum testosterone levels, the Centers for Disease Control and Prevention (CDC) developed isotope dilution-liquid chromatography-tandem mass spectrometry (ID-LC-MS/MS) for routine analysis, with the lowest linearity limit of 0.75 ng/dL. More details for the laboratory methodology were provided online (https://wwwn.cdc.gov/nchs/data/nhanes/2015-2016/labmethods/TST_I_~MET_TST_EST.pdf).

### 2.4. Covariate collection

Information on covariates was collected from questionnaires, examinations, and laboratory tests. Continuous variables included poverty income ratio (PIR), body mass index (BMI), energy, healthy eating index-2015 (HEI-2015), testosterone, estrogen, and sex hormone-binding globulin (SHBG). Categorical variables included age (≥20 years), race, education level, marital status, smoking, alcohol, components of circadian syndrome diagnosis, cancer, gout, stroke, cardiovascular disease (CVD), vigorous activity, and moderate activity (all no/yes). Estradiol and SHBG were categorized according to the tertile distribution. The data for both variables were defective in the 2011–2012 cycle, so missing data were present. Specifically, age was stratified as 20–34, 35–49, 50–64, and ≥65 years. PIR was grouped as ≤ 1.3, >1.3, ≤ 3.5, and >3.5%. Races included Mexican Americans, other Hispanics, non-Hispanic black, non-Hispanic white, and other races. Smoking included never (< 100 cigarettes in life), former (≥100 cigarettes in life and smoking not at all now), and now (≥100 cigarettes in life and smoking some days or every day in life). Alcohol was defined as < 12 or ≥12 drinks per year. Marital status was divided into married, widowed, divorced, separated, never married, and living with a partner. The education level was classified into less than 9th grade, 9th−11th grade, high school graduate, some college, and college graduate or above.

### 2.5. Statistical analysis

Mean ± standard deviation (SD) and frequency (proportions) were fully used to describe the baseline characteristics of all participants. Linear regression was used to correlate continuous variables, while the chi-square test was used to compare categorical variables. For missing information on SHGB and estrogen, dummy variables were used to indicate missing covariate values.

A total of two groups were formed based on the CircS diagnosis, and three multivariable logistic regression models were built to analyze the correlations between the components of CircS and the prevalence rates of TD. In the non-adjusted model, no factor was adjusted. The minimally adjusted model was only adjusted for age and race. The fully adjusted model was further adjusted for education, BMI, marital status, PIR, smoking, alcohol, gout, stroke, vigorous activity, moderate activity, energy, CVD, SHBG, estrogen, and total HEI-2015 score. Interactive and stratified logistic regression models were conducted regarding all potential confounding factors mentioned above in order to assess the relationship between the prevalence rates of CircS and TD more effectively. Sensitivity analyses with extreme values excluded (testosterone content >mean+3SD or < mean-3SD) were conducted to verify the stability of the results.

For all analyses, the sample weights of three continuous cycles were combined according to the method recommended by CDC (https://www.cdc.gov/nchs/nhanes/index.htm). Statistical analyses were all performed on R 4.0 (http://www.R-project.org, the R Foundation) and EmpowerStats (http://www.empowerstats.com, X&Y Solutions, Inc.). Differences were considered statistically significant when *P*-value was < 0.05.

## 3. Results

### 3.1. Baseline characters of the population

A total of 3,436 eligible participants were involved in the NHANES 2011–2016 cycles. As shown in Table 1, the population was divided into two groups by TD. Significant differences were found between the groups in the majority of the baseline characteristics. There were 841 people with TD and 2,595 people without it. People with TD were older (53.0 ± 16.2 years) and more likely to have a high BMI (33.0 ± 7.1 kg/m^2^), and particularly, obesity (BMI ≥30 kg/m^2^, 60.4%) accounted for a larger volume than other groups. Additionally, the proportion of TD participants suffering from gout (9.2%) was significantly higher. The same trend was seen in the TD group with CVD (17.1%) and CircS (57.9%). Notably, four or five diagnostic components of CircS were major parts in the TD group (29.5% and 23.6%, respectively), except for the case of fewer than four components. However, the proportions of alcohol drinking (84.1%), vigorous activity (23.8%), moderate activity (40.2), and non-smoking (never smoking, 43,4%) were lower compared with non-TD people. Moreover, lower concentrations of testosterone (225.2 ± 63.1 ng/dl), SHBG (30.3 ± 17.6 nmol/L), and estradiol (21.9 ± 8.2 pg/mL) were found.

**Table 1:** Characteristics of participants by categories of testosterone deficiency: NHANES 2011–2016^*^.

**Variables**	**All (*n* = 3,436)**	**Groups**		***P*-value**
		**Non-testosterone deficiency (*****n*** = **2,595)**	**Testosterone deficiency (*****n*** = **841)**	
Age (years, mean ± SD)	48.9 ± 16.7	47.7 ± 16.7	53.0 ± 16.2	< 0.001
20–34 (%)	24.2	26.7	15.6	
35–49 (%)	26.5	26.6	26.2	
50–64 (%)	28.5	27.7	31.6	
≥65 (%)	20.7	19.1	26.6	
BMI (kg/m^2^, mean ± SD)	29.1 ± 6.1	28.1 ± 5.3	33.0 ± 7.1	< 0.001
< 25 (%)	24.3	28.5	9.7	
25–30 (%)	37.6	40.0	29.9	
≥30 (%)	37.8	31.5	60.4	
PIR (mean ± SD)	3.0 ± 1.6	3.0 ± 1.6	3.0 ± 1.6	0.601
≤ 1.3 (%)	20.6	20.7	20.2	
>1.3 and ≤ 3.5 (%)	35.7	35.2	37.3	
>3.5 (%)	43.7	44.1	42.6	
Race (%)				0.969
Mexican American	8.7	8.9	8.2	
Other Hispanics	6.2	6.2	6.2	
Non-Hispanic white	68.9	68.7	69.3	
Non-Hispanic black	8.8	8.7	9.1	
Other races	7.5	7.5	7.2	
Education (%)				0.931
Less than 9th grade	5.2	5.2	5.4	
9-11th grade	11.3	11.3	11.4	
High school graduate	22.3	22.2	22.7	
Some college	30.1	29.9	30.9	
College graduate or above	31.0	31.4	29.7	
Marital (%)				< 0.001
Married	59.2	57.5	65.1	
Widowed	2.6	2.5	2.8	
Divorced	8.9	8.6	9.8	
Separated	1.8	1.7	1.9	
Never married	19.1	20.5	14.1	
Living with partner	8.5	9.2	6.2	
Alcohol (%)				0.018
No	13.3	12.5	15.9	
Yes	86.7	87.5	84.1	
Gout (%)				< 0.001
No	94.7	95.8	90.8	
Yes	5.3	4.2	9.2	
Vigorous activity (%)				< 0.001
No	70.8	69.2	76.2	
Yes	29.2	30.8	23.8	
Moderate activity (%)				0.002
No	54.9	53.5	59.8	
Yes	45.1	46.5	40.2	
Smoking (%)				< 0.001
Never	47.1	48.1	43.4	
Former	31.9	29.4	40.5	
Now	21.1	22.5	16.1	
Stroke (%)				0.954
No	96.9	96.9	96.9	
Yes	3.1	3.1	3.1	
CVD (%)				< 0.001
No	88.6	90.3	82.9	
Yes	11.4	9.7	17.1	
Circadian syndrome (%)				< 0.001
No	66.2	73.1	42.1	
Yes	33.8	26.9	57.9	
Diagnostic components of circadian syndrome (%)				< 0.001
< 4	66.2	73.1	42.1	
4	18.9	15.9	29.5	
5	13.1	10.1	23.6	
6	1.6	0.9	4.2	
7	0.2	0.1	0.6	
SHBG (nmol/L)	44.3 ± 24.0	47.8 ± 24.1	30.3 ± 17.6	< 0.001
≤ 31.4 (%)	21.2	17.0	36.0	
>31.4 and ≤ 50.0 (%)	20.8	22.2	15.9	
>50.0 (%)	20.3	24.6	4.9	
Missing (%)	37.8	36.2	43.2	
Estradiol (pg/mL)	26.2 ± 9.6	27.3 ± 9.6	21.9 ± 8.2	< 0.001
≤ 21.4 (%)	21.4	18.3	32.6	
>21.4 and ≤ 28.9 (%)	22.9	24.5	17.4	
>28.9 (%)	22.3	25.2	11.8	
Missing (%)	33.3	32.0	38.3	
Testosterone (ng/dL)	440.2 ± 185.6	501.0 ± 162.2	225.2 ± 63.1	< 0.001
HEI-2015 (mean ± SD)	50.2 ± 13.6	50.3 ± 13.6	50.1 ± 13.8	0.817

^*^For continuous variables, the *t*-test for the slope was used in generalized linear models.

Values are expressed as mean+SD or %. SD, standard deviation; BMI, body mass index; PIR, Poverty Income Ratio; Cardiovascular disease, CVD; Sex hormone-binding globulin, SHBG; HEI-2015, Healthy Eating Index-2015.

### 3.2. Multivariate regression analysis

The effect sizes of the association between the prevalence rates of CircS and TD are presented in Table 2. The prevalence rates of CircS were positively associated with TD in the non-adjusted model (OR = 3.731, 95%CI 2.990 to 4.657), the minimally adjusted model (OR = 3.610, 95%CI 2.828 to 4.609), and the fully adjusted model (OR = 2.284, 95%CI 1.569 to 3.323) (all *P* < 0.001). The stratified analysis showed no significant correlation between the number of CircS components and the prevalence rates of TD.

**Table 2:** Association of circadian syndrome with the prevalence rates of testosterone deficiency.

**Variables**	**Non-adjusted model** ^*^		**Minimally adjusted model** ^**^		**Fully adjusted model** ^***^	
	**OR (95%CI)**	***P***	**OR (95%CI)**	***P***	**OR (95%CI)**	***P***
**Circadian syndrome**						
No	Ref		Ref		Ref	
Yes	3.731 (2.990, 4.657)	< 0.001	3.610 (2.828, 4.609)	< 0.001	2.284 (1.569, 3.323)	< 0.001
**Components of circadian syndrome**						
4	Ref		Ref		Ref	
5	1.261 (0.856, 1.859)	0.247	1.255 (0.844, 1.867)	0.269	1.185 (0.739 1.900)	0.434
≥6	2.889 (1.243, 6.718)	0.018	2.916 (1.205, 7.052)	0.023	3.529 (0.869, 14.328)	0.069

CI, confidence interval; OR, odds ratio.

^*^Non-adjusted model adjusts for none. ^**^Minimally adjusted model adjusts for age and race. ^***^Fully adjusted model adjusts for age, race, education, BMI, marital, PIR, HEI-2015, smoking, vigorous activity, moderate activity, alcohol, gout, stroke, CVD, SHBG, and estradiol.

### 3.3. Stratified and interaction analysis

An interaction analysis was performed to further explore whether some variables can interfere with the association between the prevalence rates of CircS and TD (Table 3). We found the association was modified by moderate activity and vigorous activity. Some significantly different effects were present between people with or without activity (vigorous or/and moderate), and both of the two variables made the association more obvious. The odds ratios of moderate activity, non-moderate activity, vigorous activity, and non-vigorous activity were 1.614, 3.036, 1.338, and 2.810, respectively, with p of 0.010 and 0.021 for interaction. However, no more interaction was indicated among other factors.

**Table 3:** Logistic regression analysis to identify variables that modify the correlation between circadian syndrome and testosterone deficiency.

**Variables**	**Non-adjusted model** ^*^		**Minimally adjusted model** ^**^		**Fully adjusted model** ^***^	
	**OR (95%CI)**	**P for interaction**	**OR (95%CI)**	**P for interaction**	**OR (95%CI)**	**P for interaction**
Age (years, mean ± SD)		0.516		0.504		0.691
20–34 (%)	5.236 (2.842, 9.648)		5.302 (2.883, 9.751)		2.689 (1.218, 5.935)	
35–49 (%)	3.163 (2.109, 4.746)		3.174 (2.117, 4.760)		2.165 (1.374, 3.413)	
50–64 (%)	3.373 (2.101, 5.416)		3.408 (2.133, 5.445)		1.979 (1.105, 3.544)	
≥65 (%)	3.648 (2.714, 4.904)		3.641 (2.707, 4.896)		2.697 (1.897, 3.833)	
BMI (kg/m^2^, mean ± SD)		0.299		0.385		0.239
< 25 (%)	4.439 (2.268, 8.690)		3.917 (1.920, 7.990)		4.270 (1.876, 9.721)	
25–30 (%)	2.518 (1.727, 3.670)		2.289 (1.546, 3.390)		2.655 (1.506, 4.683)	
≥30 (%)	2.406 (1.711, 3.382)		2.262 (1.586, 3.227)		1.887 (1.223, 2.911)	
PIR (mean ± SD)		0.133		0.115		0.604
≤ 1.3 (%)	3.904 (2.719, 5.607)		3.651 (2.506, 5.319)		2.512 (1.583, 3.987)	
>1.3 and ≤ 3.5 (%)	2.733 (2.030, 3.679)		2.618 (1.932, 3.547)		1.914 (1.276, 2.871)	
>3.5 (%)	4.485 (2.780, 7.235)		4.411 (2.670, 7.289)		2.574(1.488, 4.452)	
Race (%)		0.475		0.433		0.241
Mexican American	4.304 (2.905, 6.375)		4.082 (2.776, 6.002)		3.407 (2.005, 5.790)	
Other Hispanics	3.642 (2.251, 5.893)		3.460 (2.063, 5.804)		2.654 (1.358, 5.189)	
Non-Hispanic white	3.893 (2.785, 5.441)		3.760 (2.635, 5.366)		2.307 (1.481, 3.594)	
Non-Hispanic black	3.910 (2.634, 5.804)		3.652 (2.380, 5.605)		1.800 (1.129, 2.872)	
Other races	2.302 (1.188, 4.462)		2.126 (1.126, 4.014)		1.473 (0.639, 3.396)	
Education (%)		0.162		0.132		0.292
< 9th grade	6.521 (2.792, 15.231)		6.389 (2.758, 14.801)		3.923 (1.582, 9.729)	
9–11th grade	4.171 (2.570, 6.769)		4.123 (2.469, 6.883)		1.916 (1.177, 3.117)	
High school graduate	3.019 (1.872, 4.869)		2.874 (1.737, 4.754)		2.029 (1.132, 3.636)	
Some college	2.962 (2.032, 4.319)		2.835 (1.882, 4.270)		1.844 (1.178, 2.886)	
College graduate or above	4.884 (3.020, 7.897)		4.801 (2.956, 7.796)		3.132 (1.675, 5.853)	
Marital (%)		0.091		0.090		0.147
Married	3.846 (2.845, 5.198)		3.868 (2.811, 5.321)		2.770 (1.845, 4.160)	
Widowed	0.982 (0.363, 2.657)		0.981 (0.374, 2.578)		0.410 (0.098, 1.709)	
Divorced	3.704 (1.809, 7.582)		3.733 (1.787, 7.798)		1.181 (0.509, 2.740)	
Separated	1.043 (0.278, 3.913)		1.011 (0.272, 3.760)		0.944 (0.276, 3.230)	
Never married	4.295 (2.357, 7.829)		4.263 (2.245, 8.096)		2.222 (1.060, 4.661)	
Living with partner	3.522 (1.553, 7.990)		3.486 (1.549, 7.847)		2.020 (0.737, 5.537)	
Alcohol (%)		< 0.001		< 0.001		0.268
No	4.946 (2.825, 8.659)		4.715 (2.652, 8.381)		3.236 (1.655, 6.329)	
Yes	3.546 (2.812, 4.473)		3.444 (2.667, 4.448)		2.162 (1.519, 3.078)	
Gout (%)		0.129		0.155		0.205
No	3.789 (2.996, 4.791)		3.715 (2.897, 4.764)		2.400 (1.701, 3.385)	
Yes	1.788 (0.710, 4.502)		1.830 (0.713, 4.696)		1.202 (0.442, 3.266)	
Vigorous activity (%)		0.008		0.007		**0.021**
No	4.358 (3.366, 5.643)		4.287 (3.210, 5.726)		2.810 (1.890, 4.177)	
Yes	2.225(1.475, 3.356)		2.156 (1.422, 3.271)		1.338 (0.797, 2.247)	
Moderate activity (%)		< 0.001		< 0.001		**0.010**
No	4.870 (3.839, 6.178)		4.794 (3.711, 6.194)		3.036 (2.080, 4.432)	
Yes	2.576 (1.862, 3.565)		2.486 (1.761, 3.511)		1.614 (1.053, 2.472)	
Smoking (%)		0.152		0.123		0.062
Never	4.466 (3.096, 6.444)		4.492 (3.084, 6.543)		3.057 (1.946, 4.803)	
Former	3.281 (2.266, 4.751)		3.333 (2.298, 4.835)		2.012 (1.298, 3.118)	
Now	2.437 (1.442, 4.118)		2.363 (1.357, 4.114)		1.372 (0.698, 2.697)	
Stroke (%)		0.776		0.730		0.532
No	3.802 (3.031, 4.769)		3.664 (2.854, 4.703)		2.260 (1.632, 3.130)	
Yes	4.564 (1.279, 16.288)		4.583 (1.269, 16.546)		3.606 (0.787, 16.533)	
CVD (%)		0.298		0.329		0.217
No	3.774 (2.858, 4.984)		3.711 (2.769, 4.973)		2.416 (1.705, 3.423)	
Yes	2.549 (1.381, 4.705)		2.574 (1.395, 4.750)		1.462 (0.694, 3.077)	
SHBG (nmol/L)		0.640		0.527		0.302
≤ 31.4 (%)	4.829 (3.153, 7.398)		3.818 (2.448, 5.955)		3.102 (1.819, 5.290)	
>31.4 and ≤ 50.0 (%)	4.819 (2.637, 8.807)		3.551 (1.971, 6.399)		2.658 (1.275, 5.539)	
>50.0 (%)	5.354 (2.729, 10.501)		4.443 (2.274, 8.678)		3.123 (1.468, 6.645)	
Missing (%)	3.636 (2.582, 5.121)		2.749 (1.891, 3.996)		1.688 (1.092, 2.610)	
Estradiol (pg/mL)		0.339		0.305		0.255
≤ 21.4 (%)	4.132 (2.526, 6.760)		4.038 (2.431, 6.709)		2.568 (1.417, 4.656)	
>21.4 and ≤ 28.9 (%)	5.932 (3.759, 9.361)		5.852 (3.672, 9.325)		4.114 (1.933, 8.755)	
>28.9 (%)	3.081 (1.830, 5.188)		3.035 (1.795, 5.133)		2.054 (1.151, 3.667)	
Missing (%)	3.663 (2.518, 5.329)		3.569 (2.414, 5.276)		1.704 (1.087, 2.672)	

CI, confidence interval; OR, odds ratio.

^*^Non-adjusted model adjusts for none. ^**^Minimally adjusted model adjusts for age and race. ^***^Fully adjusted model adjusts for age, education, race, BMI, marital, PIR, gout, HEI-2015, smoking, vigorous activity, moderate activity, alcohol, stroke, CVD, SHBG, and estradiol.

### 3.4. Sensitivity analyses

Sensitivity analyses were conducted to evaluate the effects of extreme values of testosterone on the results (Supplementary Table S1), which showed that the positive association between the prevalence rates of CircS and TD was still present. A higher prevalence rate of CircS was correlated with a higher risk of TD. Additionally, the association was not disturbed by the number of CircS components.

## 4. Discussion

This cross-sectional study encompassed a total of 3,436 eligible participants and relied on the data extracted and analyzed from three cycles of NHANES. The results showed that the prevalence rates of CircS were positively associated with TD, with no modifications observed in the association's severity due to CircS. Additionally, moderate or vigorous activities made the positive relationship more obvious. With extreme values of testosterone excluded, the sensitivity analyses showed the same result. Therefore, we believe that the results are robust.

Previous research has established a discernible rhythm in testosterone secretion, with peak secretion occurring between 8 a.m. and 9 a.m. Disturbances to the circadian rhythm can result in diurnal variations in testosterone (7). A multitude of studies was published on the association between TD and many diseases, such as metabolic diseases, depression, or short sleep duration. However, these diseases were always analyzed alone and never considered at the same time. Therefore, this is the first study to demonstrate the positive correlation between the prevalence rates of TD and CircS. As CircS is a relatively new concept, only circumstantial evidence supports the positive relationship between the prevalence rates of CircS and TD. CircS is mainly controlled by circadian rhythm dysfunction, which refers to a set of biological variables produced by biological clocks within 24 h (10). The circadian clock not only regulates the daily physiological and molecular rhythms but also rhythmically controls gene expressions (19). The dissonance of the biological clock and endocrine is the primary cause of circadian rhythm disorders. When dysfunctions occur, physiological and pathological disorders such as MetS, cardiovascular diseases, and neuropsychiatric disorders may develop (11, 20). All of these factors mentioned above can contribute to TD development (21, 22). Recent studies suggest that circadian rhythms are primarily controlled by the suprachiasmatic nucleus (SCN) of the hypothalamus, regardless of peripheral clocks. Moreover, changes in the master body clock are primarily induced by light intensity and duration (23). Shift workers, people who stay up late, and people who sleep little are more prone to circadian rhythm disorders and TD (24, 25). Recent rat experiments support that short sleep can induce an intense alternation in the hypothalamic-pituitary-adrenal axis, resulting in elevated serum cortisol levels and reduced testosterone levels (26, 27). In detail, the increased cortisol content significantly decreases testosterone levels *via* direct access (e.g., its receptors in Leydig cells) or by inducing Leydig cell apoptosis (28, 29). Thus, maintaining a regular and healthy schedule to ensure circadian rhythms, especially later in life, may not only decrease the risk of CircS and TD but also improve overall happiness and quality of life.

Testosterone secretion mainly depends on the hypothalamus-pituitary-gonadal (HPG) axis. Gonadotropin-releasing hormone (GnRH), at the upstream of the HPG axis, is produced in the hypothalamus and stimulates testosterone production (30). In addition, the release of GnRH is closely related to hypothalamic N-peptide kisspeptin encoded by the KISS1 gene and kisspeptin receptor on GnRH neurons. Research focusing on TD indicates that the transmission of blocked kisspeptin signaling plays a critical role in GnRH release (30). For obese people with CircS, increased adipokine secretion (adiponectin, leptin) can block kisspeptin signaling and inhibit GnRH release, leading to decreased testosterone secretion (31, 32). However, the relationship between leptin and testosterone is complex, as normal leptin levels can promote kisspeptin expression and testosterone synthesis, but high levels of leptin can directly inhibit Leydig cells by decreasing 17, 20-lyase activity (33, 34). In addition, adiponectin can activate AMP-activated protein kinase (AMPK) and decrease coexisting KISS1 expression, further inhibiting GnRH release (35). Furthermore, there is a bidirectional regulatory relationship between TD and adipokine. In the presence of TD, the inhibitory effects of adipocyte-produced adiponectin and the contribution to leptin concentration diminished (36, 37). Therefore, monitoring and adjusting adipokine content may be important in the prevention and treatment of TD and CircS.

As diet and lifestyle change, circadian rhythm disorders occur more easily and are accompanied by metabolic diseases, such as diabetes, insulin resistance, hyperlipidemia, and HBP (16, 38–40). Studies show that these diseases can have negative impacts on testosterone secretion in men, especially for those who are obese and insulin-resistant (41). Circadian rhythm dysfunction promotes the secretion of inflammatory cytokines (TNFα, IL-1β, and IL-6), which induces insulin resistance and makes a negative effect on the production of GnRH and LH (42–44). Moreover, mitochondrial and endoplasmic reticulum are essential factors in guaranteeing testosterone synthesis in Leydig cells (45). Hyperlipidemia can induce impaired lipid profile and ROS production, leading to the overproduction of mitochondrial ROS and disturbing endoplasmic reticulum protein folding, which can damage mitochondrial structure impairment and endoplasmic reticulum stress (46, 47). These results support that the free-cholesterol level increases and testosterone synthesis declines in Leydig cells. Moreover, HBP causes less blood supply to Leydig cells through small arterial vasculopathy (testicular arteries), which further negatively impacts testosterone synthesis (48). However, the exact mechanisms behind the phenomenon still remain vague.

According to the stratified and interaction analyses, moderate and vigorous activities can modify the positive association between the prevalence rates of CircS and TD, and the results are even more pronounced for people without moderate or vigorous activities. A randomized controlled trial indicates that both the serum and testis testosterone concentrations increase significantly following exercise (49). Numerous analyses support that exercise not only resets the clock in patients with metabolic diseases, depression, or short sleep but also reinforces endogenous circadian rhythms (50, 51). As such, exercise may serve as a pivotal intervention in the prevention and treatment of CircS and TD.

This study included a representative sample of the multiracial population in the United States, and the large populations allowed further subgroup and interaction analyses. This is the most outstanding aspect of the design. Furthermore, the concept of CircS combines with biological rhythms that are more consistent with the majority of the physiological processes. To the best of our knowledge, no study has focused on the association between the prevalence rates of CircS and TD. However, some limitations must also be acknowledged. First, the cross-sectional design of the study did not elucidate the causality between the prevalence rates of CircS and TD. Second, fasting serum for 1 day was only provided, and two fasting samples from the same participant for testosterone deficiency diagnosis were unavailable. In addition, despite the adjustment for many study-relevant confounding factors, we cannot exclude all of them, especially the co-exposed factors. Therefore, further comprehensive research is needed to elucidate the causal relationship between the prevalence rates of CircS and TD.

## 5. Conclusion

There is a positive association between the prevalence rates of CircS and TD, and the association is robust and independent of the number of CircS components. The positive association becomes more obvious in the case of moderate or vigorous activities.

## Data availability statement

The datasets presented in this study can be found in online repositories. The names of the repository/repositories and accession number(s) can be found below: https://www.cdc.gov/nchs/nhanes/index.htm, National Center for Health Statistics.

## Ethics statement

This study was performed using public data from the National Center for Health Statistics (NCHS) program and the National Health and Nutrition Examination Survey (NHANES). The data have been de-identified and not merged or augmented in any way that has compromised the privacy of the participants. Therefore, the study requires no further approval and follows ethical guidelines.

## Author contributions

JiaW and SY: conception and design. JiaW: administrative support and supervision. YX, JiahW, JC, and YB: collection and assembly of data. YX, SY, JC, and ZY: data analysis and interpretation. YX: manuscript writing. All authors approved the final version of the manuscript.
