# Supplementary material for: Association between the prevalence rates of circadian syndrome and testosterone deficiency in US males: data from NHANES (2011–2016)
Source: Front Nutr. 2023 May 9;10:1137668. doi: 10.3389/fnut.2023.1137668 (PMC10204805; doi:10.3389/fnut.2023.1137668)
Supplement: Supplementary file 1 [file Table_1.docx]

| **Variables** | **Non-adjusted model*** | | **Minimally adjusted model**** | | **Fully adjusted model***** | |
| --- | --- | --- | --- | --- | --- | --- |
|  | **OR (95%CI)** | **P** | **OR (95%CI)** | **P** | **OR (95%CI)** | **P** |
| **Circadian syndrome** |  |  |  |  |  |  |
| No | Ref |  | Ref |  | Ref |  |
| Yes | 3.705 (2.966, 4.630) | <0.001 | 3.578 (2.801, 4.572) | <0.001 | 2.263 (1.554, 3.295) | <0.001 |
| **Components of circadian syndrome** |  |  |  |  |  |  |
| 4 | Ref |  | Ref |  | Ref |  |
| 5 | 1.271 (0.864, 1.870) | 0.230 | 1.265 (0.853, 1.877) | 0.249 | 1.199 (0.763 1.884) | 0.384 |
| ≥6 | 2.879 (1.238, 6.695) | 0.018 | 2.906 (1.202, 7.025) | 0.023 | 3.421 (0.872, 13.414) | 0.069 |

CI: confidence interval, OR: odds ratio

*Non-adjusted model adjusts for none. 
** Minimally adjusted model adjusts for age, race. 
*** Fully adjusted model adjusts for age, race, education, BMI, marital, PIR, HEI-2015, smoking, vigorous activity, moderate activity, alcohol, gout, stroke, CVD, SHBG, estradiol.

**Table S1** Association of circadian syndrome with the prevalence of testosterone deficiency with extreme values of testosterone removed.
